# A review of risk factors and intervention measures for comorbidity of diabetes and mental disorders

**DOI:** 10.3389/fpsyt.2026.1701730

**Published:** 2026-04-10

**Authors:** Mu Chen Zhou, Tai Hong Zhao

**Affiliations:** 1 The Affiliated Brain Hospital of Nanjing Medical University, Nanjing, Jiangsu, China; 2 Office of the Dean, The Affiliated Brain Hospital of Nanjing Medical University, Nanjing, Jiangsu, China

**Keywords:** comorbidity management, diabetes mellitus, intervention measures, mental disorders, pathogenesis

## Abstract

Globally, the increasing prevalence of diabetes and mental disorders coexisting as comorbid conditions poses significant challenges for chronic disease management. The interaction mechanisms between these two conditions involve multiple pathways, including neuroendocrine dysregulation, chronic inflammation, and neurotransmitter abnormalities. This comorbidity severely impacts patients’ health outcomes and increases clinical management complexity. Based on a narrative synthesis of relevant literature, this paper reviews the epidemiological evidence and explores the bidirectional mechanisms linking these conditions across biological, behavioral, and healthcare system levels. It further summarizes integrated management models and tiered intervention strategies. The aim is to provide a theoretical foundation and practical guidance for establishing a more effective prevention and treatment system for this psychosomatic comorbidity.

## 1 Introduction

Diabetes mellitus is a metabolic disorder characterized by chronic hyperglycemia. Its global prevalence continues to rise, making it one of the most severe public health challenges of the 21st century. According to the International Diabetes Federation (IDF), the number of people with diabetes worldwide reached 463 million in 2019 and is projected to increase to 780 million by 2045 (1). Diabetes not only increases the risk of organic complications but also frequently coexists with various mental disorders, exacerbating patients’ burdens and mortality risk.

Mental disorders refer to a range of cognitive, emotional, and behavioral disturbances caused by abnormal brain function. In this review, we primarily focus on mood disorders (major depressive disorder and bipolar disorder), anxiety disorders, and schizophrenia spectrum disorders. Neurocognitive disorders, including cognitive impairment and dementia, are discussed only insofar as they represent metabolic–neuropsychiatric consequences of diabetes rather than primary psychiatric conditions within the scope of this review. They exhibit epidemiological characteristics such as high prevalence, prolonged course, and widespread comorbidity. In recent years, the interrelationship between mental disorders and chronic metabolic diseases—such as diabetes, metabolic syndrome, and thyroid dysfunction—has garnered increasing attention (2). However, the comprehensive management of mental disorders still faces multiple challenges: at the health system level, mental health resources remain severely underfunded, primary-level services operate with low efficiency, and early identification and intervention systems remain underdeveloped (3). At the patient level, widespread stigma associated with mental illness and a lack of self-management awareness negatively impact treatment-seeking rates and adherence (4). At the clinical level, standardized guidelines and individualized treatment strategies for the co-occurrence of mental disorders and chronic physical illnesses have yet to be established (5). These factors collectively constrain the early identification, precise treatment, and ongoing management of individuals with mental disorders.

A growing body of research confirms a complex bidirectional relationship between diabetes and mental disorders: On one hand, individuals with mental disorders, due to prolonged stress states, experience hyperactivity of the HPA axis, activation of inflammatory responses, and increased oxidative stress, making them susceptible to insulin resistance and metabolic abnormalities (6). On the other hand, the chronic course of diabetes, adverse life events, and poor treatment adherence also promote the onset and exacerbation of negative emotions such as depression and anxiety (7). This comorbidity significantly increases the complexity of clinical treatment, posing a major challenge for lifelong health management.

In recent years, lifestyle adjustments and the widespread adoption of digital healthcare services have enhanced the general population’s capacity for diabetes prevention and management, leading to a declining trend in diabetes incidence rates in some regions (8). However, individuals with mental disorders have not benefited equally from these interventions, and their risk of developing diabetes remains persistently high. Similarly, the prevalence of mental disorders among people with diabetes has not decreased as a result. Studies indicate that individuals with serious mental illness have a 2–3 times higher prevalence of diabetes than the general population, with an average reduction in life expectancy of 10–15 years (9). The incidence of depressive disorders among people with diabetes is approximately 2–3 times higher than in the general population (10). Among individuals with type 1 diabetes (T1DM), approximately 14% experience comorbid mental disorders, including depression, anxiety, mood disorders, and post-traumatic stress disorder (11). In a retrospective analysis of psychiatric outpatient treatment, Lamberti et al. identified a significant comorbidity between schizophrenia and diabetes, potentially attributable to medication use. Among schizophrenia patients treated with atypical antipsychotics, the prevalence of diabetes reached 14.2%, markedly higher than that observed in the general population (12). Furthermore, Chan JKN et al. observed increased incidence and mortality rates of diabetes among schizophrenia patients, with gender and age emerging as significant risk factors for this comorbidity (13). Collectively, these findings underscore the critical importance of managing the co-occurrence of mental disorders and diabetes.

This narrative review examines the potential mechanisms underlying the comorbidity of mental disorders and diabetes, synthesizes evidence on current intervention and management strategies, and proposes future directions to optimize health outcomes for patients with comorbid conditions. Beyond existing reviews, this paper contributes a tiered intervention model that integrates psychometabolic screening, personalized pharmacotherapy, and digitally-supported collaborative care, offering a practical framework for clinical implementation.

## 2 Methods

This article is a narrative review. To identify relevant literature, searches were conducted in PubMed and Web of Science for articles published up to October 2025. Search terms included combinations of: (“diabetes mellitus” OR “T2DM”) AND (“mental disorders” OR “depression” OR “anxiety” OR “schizophrenia” OR” Bipolar Disorder”) AND (“comorbidity” OR “co-occurrence”) AND (“mechanism” OR “pathophysiology” OR “risk factor”) AND (“management” OR “intervention”). Reference lists of key articles were also screened. The included literature is categorized by topic into epidemiological studies, mechanistic studies (clinical and preclinical), clinical trials, and review articles published in English. Inclusion criteria were: (1) studies examining the association, mechanisms, or management of diabetes-mental disorder comorbidity; (2) human studies (with preclinical mechanistic studies included for biological plausibility); (3) systematic reviews and meta-analyses prioritized for epidemiological estimates. Exclusion criteria were: (1) case reports, case series, and conference abstracts; (2) non-English publications;

## 3 Evidence synthesis

### 3.1 Epidemiology

The comorbidity between diabetes and mental disorders is highly prevalent and bidirectional, with substantial evidence demonstrating that the presence of one condition significantly increases the risk of developing the other. Epidemiological studies have consistently quantified this association across diverse populations and mental disorder subtypes (**Table 1**).

**Table 1:** Epidemiological studies examining the bidirectional relationship between diabetes and mental disorders.

Association	Type	Key finding	Population	Sample	Effect measure (95% CI)	Ref
Depression + Diabetes Complications	Prospective cohort	Increased risk of microvascular and macrovascular complications	T2DM patients	6019	HR 1.37 (1.13–1.65)	(15)
PTSD + T1DM	Observational	PTSD associated with worse T1DM outcomes	T1DM patients	152,265		(11)
Schizophrenia+ Diabetes	Population-based cohort	Increased mortality and complications	Schizophrenia with diabetes	75,673	aHR 1.11 (CI 1.05–1.18)	(13)
Severe Mental Illness → T2DM	Register-based cohort	Higher diabetes complication rates	SMI patients	371625		(39)
Schizophrenia →T2DM	Cohort study	Psychiatric symptoms predicted T2DM onset	US Veterans	543979	OR = 1.30 ( 1.21 - 1.40)	(28)
T2DM + SMI	Longitudinal study	Increased healthcare use & poorer outcomes	England primary care	9965	HR=1.919 (1.60 - 2.30)	(58, 59)
Chronic conditions → Perinatal mental illness	Population cohort	Physical illness increases psychiatric risk	Perinatal population	858004	AR= 1.20 (1.18 - 1.22)	(5)

### 3.2 Biological mechanisms

#### 3.2.1 Neuroendocrine axis dysregulation

The hypothalamic-pituitary-adrenal (HPA) axis is a critical neuroendocrine system that regulates stress responses, emotional states, the immune system, energy metabolism, and other physiological processes (14). Excessive activation of the HPA axis leads to abnormal cortisol secretion (elevated basal levels and disrupted circadian rhythms), impairing insulin sensitivity and inducing insulin resistance (15). Concurrently, excessive cortisol suppresses hippocampal neuroplasticity, compromising emotional functioning and triggering affective symptoms such as anxiety and depression. If unmanaged, this can further precipitate the onset of psychiatric disorders (16). Furthermore, brain insulin resistance, a consequence of systemic insulin resistance in diabetes, may reduce clearance of β-amyloid (Aβ), linking it to cognitive impairment pathways (17, 18). Human prospective evidence: moderate; mechanistic evidence: strong; RCT evidence: limited.

#### 3.2.2 Oxidative stress and inflammatory response

Chronic hyperglycemia drives excessive reactive oxygen species (ROS) production, disrupting oxidative homeostasis. ROS contribute to forming advanced glycation end-products (AGEs) (19). AGEs, via receptor (RAGE) binding, can promote β-cell apoptosis (24, 25) and, upon crossing the blood-brain barrier, damage neurons and disrupt neurotransmitter synthesis (20–23), forming a basis for psychiatric symptoms.

ROS also activate nuclear factor kappa-B (NF-κB), leading to the release of pro-inflammatory cytokines like IL-6 and TNF-α (26). Peripherally, these cytokines inhibit insulin receptor substrate-1 (IRS-1) phosphorylation, inducing insulin resistance. Centrally, they trigger neuroinflammation, damaging brain regions involved in mood and cognition (27). Notably, patients with mood disorders or schizophrenia often show elevated pro-inflammatory markers (28), which may exacerbate metabolic dysfunction (29), creating a vicious cycle. However, findings on specific inflammatory biomarkers (e.g. IL-6, CRP) across different comorbid populations are sometimes inconsistent, potentially due to heterogeneity in study designs, patient characteristics, or measurement timing, highlighting a need for standardized longitudinal assessments. Human prospective evidence: moderate; mechanistic evidence: strong; RCT evidence: limited (for anti-inflammatory interventions).

#### 3.2.3 Abnormal neurotransmitter metabolism

Neurotransmitters such as dopamine (DA) and serotonin (5-HT) not only directly regulate mental activities like mood and cognition but also participate in energy metabolism and insulin sensitivity regulation through central and peripheral mechanisms. For instance, diminished 5-HT receptor function and imbalanced dopamine and glutamate signaling in the prefrontal cortex contribute to the negative symptoms of schizophrenia (30). Additionally, this dysregulation affects the HPA axis and leptin signaling, leading to abnormal appetite and obesity, subsequently inducing insulin resistance (31). Under hyperglycemic conditions, the glycolytic byproduct methylglyoxal (MGO) accumulates extensively. It reacts with dopamine to generate neurotoxic metabolites, damaging the substantia nigra-striatal pathway and inducing Parkinson-like pathological changes. It may be a cause of cognitive impairment and depression (32).

### 3.3 Behavioral and psychological factors

Unhealthy lifestyles such as high-fat diets, physical inactivity, and smoking are significant risk factors for the co-occurrence of diabetes and mental disorders. High-fat diets not only promote insulin resistance through fat deposition but also accelerate Aβ deposition and excessive phosphorylation of tau protein in the brain by activating inflammation-related pathways, thereby inducing neurodegenerative changes and cognitive decline (33). High-salt diets activate the HPA axis, increasing cortisol secretion and inducing anxiety and depressive moods (34). Additionally, diet-induced brain insulin resistance reduces Aβ clearance, exacerbating neurodegeneration. However, studies indicate that dietary interventions can partially reverse these pathological processes (35).

Lack of regular exercise reduces endorphin levels, potentially impairing emotional regulation. Prolonged sedentary behavior decreases the synthesis of monoamines like serotonin and dopamine, triggering metabolic disorders and increasing risks of hyperglycemia and depression (36, 37). Exercise also modulates neurotrophic factors and inflammatory states, making it a key strategy for improving T2DM-related cognitive impairment (38).

Smoking is more prevalent among individuals with severe mental illness (SMI). Research indicates that this population often exhibits poor dietary habits and insufficient physical activity, contributing to impaired glycemic control and heightened risk of diabetic complications (39).

Poor treatment adherence also increases the risk of diabetes-mental disorder comorbidity. On the physiological level, poor medication adherence can directly lead to large fluctuations in blood sugar or insufficient emotional regulation, thereby promoting the pathogenesis of comorbidities (40). At the psychosocial level, inadequate treatment adherence is often closely linked to weak social support systems. Factors such as lack of family support, poor doctor-patient communication, or financial constraints reduce patients’ motivation for self-management and adherence to treatment plans (41, 42).

These combined factors contribute to metabolic disorders and neurological damage, ultimately driving the pathophysiological progression of diabetes-mental disorder comorbidity.

### 3.4 Pharmacological factors

#### 3.4.1 Psychotropic drugs

Antipsychotic drugs are classified into typical and atypical antipsychotics based on their mechanisms of action on dopamine and serotonin receptors. Atypical antipsychotics are more likely to cause metabolic abnormalities or weight gain, thereby increasing the risk of diabetes onset (43). While atypical antipsychotics are widely used to treat severe mental disorders, concerns about their metabolic side effects are growing. A prospective cohort study by Miyakoshi T et al. demonstrated that SGAs significantly increase the risk of diabetes and metabolic syndrome, particularly clozapine, olanzapine, and quetiapine, while risperidone and amisulpride carry lower risks (44, 45). Research indicates that olanzapine not only induces metabolic disorders such as increased appetite, insulin resistance, and dyslipidemia by antagonizing 5-HT2C receptors but also directly suppresses insulin secretion from β-cells, leading to unstable blood glucose levels (46). Furthermore, Certain antidepressants alleviate depressive symptoms by regulating neurotransmitter balance in the body. For example, venlafaxine improves depressive symptoms by influencing levels of both serotonin and norepinephrine (47); however, its effects on the endocrine system increase patients’ risk of developing diabetes.

#### 3.4.2 Diabetes medications

Commonly used hypoglycemic agents include biguanides, alpha-glucosidase inhibitors, sulfonylureas, glinides, thiazolidinediones, DPP-4 inhibitors, GLP-1 receptor agonists, SGLT-2 inhibitors, insulin preparations, and traditional Chinese patent medicines (48). Among these, sulfonylureas and glinides act by binding to the sulfonylurea receptor on pancreatic β-cell surfaces, potentially inducing hypoglycemia. This binding closes ATP-dependent potassium channels. Following membrane depolarization, calcium ions influx, promoting insulin secretion. This mechanism is independent of glucose levels, carrying a higher risk of hypoglycemia (49, 50). Research indicates that recurrent hypoglycemia can impair cognitive function in diabetic patients, potentially leading to Alzheimer’s disease and cognitive impairment (51). A Canadian cohort study by Haroon NN et al. demonstrated that hypoglycemia is the most significant risk factor for dementia in elderly diabetic patients (52).

### 3.5 Factors within the healthcare system

#### 3.5.1 Insufficient collaboration between psychiatric and chronic disease departments

Patients with comorbid mental disorders and diabetes often face fragmented care and a lack of effective communication and coordination, limiting management outcomes and significantly reducing the efficiency of comprehensive disease management (53). The current healthcare system exhibits a disconnect between mental health and physical health services. This forces patients with diabetes and mental disorders to navigate two separate healthcare systems. The lack of electronic medical record sharing and referral mechanisms means only a small proportion of Medicaid beneficiaries with SMI receive standardized chronic disease risk screening and management services (54). Second, the specialization of medical education systems leaves psychiatrists lacking experience in chronic disease management, while internists cannot recognize and intervene in psychiatric symptoms, further hindering collaboration (55). Unclear delineation of responsibilities between psychiatric and chronic disease departments creates gaps in patient management. Implicit biases among healthcare providers may also reduce physicians’ vigilance and willingness to intervene regarding patients’ cardiovascular and metabolic risks (56). Therefore, establishing efficient collaboration mechanisms between psychiatric and chronic disease departments, promoting interoperability of electronic health records, optimizing referral processes, and enhancing clinical training on integrated comorbidity management are critical measures to improve health outcomes for patients with coexisting mental disorders and diabetes.

#### 3.5.2 Absence of integrated care models

Currently, the management of patients with diabetes and severe mental illness (SMI) generally lacks an “integrated care model”—a coordinated system for managing both physical and mental health conditions. This absence has become a significant factor affecting patient health outcomes and increasing healthcare costs. Research by Magaard JL et al. indicates that within the German healthcare system, mental diagnoses overshadow attention to physical conditions, leading to inefficient diagnosis of physical diseases like diabetes among SMI patients. This results in delayed diagnosis and inadequate treatment (57). Although policies like the UK’s Quality and Outcomes Framework (QOF) have improved overall diabetes care quality, the SMI population still lacks individualized interventions and disease-specific co-management pathways. SMI patients consistently show disadvantages in indicators such as metabolic monitoring, cardiovascular disease screening, and diabetes-related hospitalization rates (58).

Multinational studies further reveal that individuals with schizophrenia are particularly vulnerable to discontinuities in primary care and specialist interventions due to cognitive impairments and social functional limitations. Despite significantly increased healthcare resource utilization, this fails to translate into improved diabetes care quality, highlighting that the current department-centric, fragmented management model cannot meet the complex needs of SMI+T2DM patients (59–61). Research consistently advocates for establishing multidisciplinary team collaboration models encompassing primary care, psychiatry, and endocrinology, alongside policy, funding, and information system support for “dual management of physical and mental health” to improve health outcomes and survival rates for this high-risk population (62). Studies indicate severely inadequate monitoring of risk factors during treatment for comorbid patients. For instance, Morrato et al. found that fewer than 20% of SGA patients underwent baseline blood glucose testing, while less than 10% received baseline lipid testing (63). Jennex et al. compared rates of lipid and glucose monitoring between mental health clinic patients and HIV clinic patients, revealing significantly lower rates among the former, highlighting gaps in health management for individuals with mental disorders (64).

## 4 Intervention measures

### 4.1 Psychometabolic risk screening model

Establishing a psychometabolic risk screening model holds significant clinical value in the comprehensive management of patients with diabetes and mental disorders. Due to limitations in human and resource capacity for comprehensive psychological screening, clinicians require an efficient, simple, and practical screening tool (65).

Recent years have witnessed the development of multiple risk prediction models aimed at identifying high-risk populations for comorbidity. These models employ diverse methodological approaches—ranging from traditional logistic regression and nomogram development to advanced deep learning architectures—and target different clinical outcomes including depression, anxiety, and cognitive impairment in diabetic populations. **Table 2** summarizes selected models that illustrate this diversity of approaches, target populations, and performance characteristics reported in the literature.

**Table 2:** Summary of some psychometabolic risk prediction models.

Model (target population)	Data Source	Predictors	Outcome	AUC (training/validation)	External validation
Depression in T2DM (Nomogram) (66)	NHANES 2007–2014 (n=4,280)	Age, gender, PIR, BMI, education, smoking, LDL-C, sleep duration, sleep disturbance	Depression	0.780/0.752	No
Depression in hospitalized T2DM (67)	Single-center China (n=308)	Gender, BMI, LDL-C, CIRS score	Depression	Not reported	No
MCI in elderly T2DM (Nomogram) (68)	Single-center China (n=306)	Education, diabetes duration, depression duration, HbA1c, walking speed, sedentary time	Mild Cognitive Impairment	0.893/Not reported	No
Depression/anxiety in T2DM (REDAPM) (69)	Nanjing regional EHR (n=24,724 internal; 34,340 external)	Multi-modal EHR data (structured + unstructured, temporal dependencies)	Depression and anxiety episodes	0.903/0.736	Yes(separate 2022 cohort)

T2DM, type 2 diabetes mellitus; PIR, poverty income ratio; LDL-C, low-density lipoprotein cholesterol; CIRS, Cumulative Illness Rating Scale; SDS, Self-Rating Depression Scale; MoCA, Montreal Cognitive Assessment; EHR, electronic health records; REDAPM, Regional Depression and Anxiety Prediction Model; AUC, area under the receiver operating characteristic curve.

Despite their potential, clinical implementation of these models faces several challenges. First, generalizability is limited—only REDAPM (69) conducted external validation, revealing a substantial AUC drop from 0.903 to 0.736, while others explicitly acknowledge lack of validation (66) or are single-center studies (67, 68). Second, missing data poses practical barriers, as models require variables not routinely available (e.g., walking speed (68), CIRS (67), detailed sleep metrics (66, 68)). Third, equity considerations demand attention—predictors may operate differently across subgroups (67), and models developed in homogeneous populations may underperform in diverse groups, potentially exacerbating health disparities. Transparent reporting (TRIPOD guidelines) and validation across diverse populations are essential before widespread implementation.

Furthermore, among patients with severe mental disorders, antipsychotic-induced metabolic disturbances significantly elevate diabetes risk. Consequently, scholars propose integrating mental disorder patients into metabolic monitoring systems and developing screening models incorporating biochemical indicators like BMI, waist-to-hip ratio, and HbA1c to enhance early co-morbidity identification (70, 71).

In summary, a multidimensional clinical psychometabolic risk prediction model can significantly improve the diagnosis rate of high-risk populations with diabetes and mental disorders, assist clinicians in developing personalized treatment and health management strategies, and provide a scientific basis for implementing precision interventions and optimizing healthcare resource allocation.

### 4.2 Regular monitoring of blood glucose, lipids, and patient psychological status

Metabolic monitoring should be integrated into the health management of patients with mental disorders. Extensive research indicates that patients taking antipsychotic medications, particularly second-generation antipsychotics (SGAs), face substantially increased risks of diabetes, dyslipidemia, and weight gain (43). Consequently, international clinical guidelines issued by the American Diabetes Association (ADA) and the American Psychiatric Association (APA) recommend conducting baseline metabolic assessments—including weight, BMI, waist circumference, fasting blood glucose, lipid profile, and blood pressure measurements—prior to initiating SGA treatment (72). Follow-up assessments should occur at weeks 4, 8, and 12 post-initiation, with quarterly monitoring thereafter. Fasting blood glucose and lipid levels should be monitored at three months, followed by annual or clinically indicated monitoring, with medication adjustments based on individual patient circumstances (73).

Mental health should be integrated into diabetes management, with psychological status assessment ongoing throughout treatment. Early screening, evaluation, and monitoring of mental health are essential, particularly for patients with a history of depression or anxiety. Special attention should be given to emotional well-being during disease progression or when other psychosocial factors arise. Regular mental health screening using scales such as the PHQ-9 and GAD-7 is recommended.

### 4.3 Psychosocial interventions combined with lifestyle management

Intervention strategies include psychotherapy and individualized lifestyle plans. Psychotherapy may integrate health education with cognitive behavioral therapy (CBT). CBT effectively alleviates depressive symptoms and improves treatment adherence by adjusting cognition and enhancing self-efficacy (74). Lifestyle interventions should advocate the Mediterranean diet pattern, which supports anti-inflammatory effects and improves insulin sensitivity. Regular exercise is recommended at 150 minutes weekly of aerobic activity or high-intensity interval training (HIIT). These dietary and exercise plans effectively reduce fat deposition and improve metabolic parameters (75). Combined interventions yield greater effects than single approaches. Opie RS et al. found CBT plus exercise activation significantly improved both metabolic and depressive symptoms (76). Dietary and exercise behaviors can be tracked via apps, enabling regular feedback mechanisms.

Interventions can be tailored to individual physiological and psychological characteristics: Adolescent patients often experience emotional instability and poor compliance during puberty. Interventions should emphasize family involvement, such as parental participation in CBT sessions (77). Older adults, facing diminished physiological function and increased risk of comorbidities, benefit more from simplified, moderate-intensity exercise forms like brisk walking and stretching. Dietary focus should include low-glycemic foods and plant-based options rich in dietary fiber (78). Concurrently, mental health concerns in this population warrant heightened attention. Beyond traditional pharmacological and psychological treatments, proactive family observation of emotional changes and patient companionship are essential components of family and social support.

### 4.4 Individualized pharmacotherapy strategies

In treating diabetes with comorbid psychiatric disorders, individualized medication selection must balance efficacy and risk while addressing both psychiatric symptom control and metabolic safety. Regarding antipsychotics, certain second-generation antipsychotics (SGAs) such as olanzapine and clozapine are closely associated with metabolic abnormalities and should be avoided in patients with metabolic comorbidities (79). In contrast, metabolically neutral agents like aripiprazole, ziprasidone, and lurasidone are more suitable for patients with diabetes or high metabolic syndrome risk (80). In diabetes management, certain hypoglycemic agents concurrently exhibit blood glucose-lowering and antidepressant effects. For example, SGLT-2 inhibitors not only offer cardiovascular and renal protection along with weight reduction benefits but have also been demonstrated to lower the risk of dementia in diabetic patients (81).

Furthermore, in cases of polypharmacy, healthcare teams must regularly assess drug interactions, patient adherence, and the risk of comorbidities. Rational drug combinations can reduce the incidence of metabolic disorders while helping stabilize mood issues in psychiatric patients.

### 4.5 Integrated management models

First, establish an integrated, patient-centered chronic disease co-morbidity management center that transcends departmental boundaries between psychiatry and internal medicine. Create a multidisciplinary team (MDT) mechanism led by the cardiovascular department of a general hospital, with collaborative participation from psychiatry, neurology, psychosomatic medicine, pharmacy, clinical nutrition, and specialists from psychiatric hospitals. Implement electronic medical record sharing and utilize early screening prediction models to identify high-risk populations for co-morbidities, enabling bidirectional referrals and remote consultations. Develop comprehensive, individualized treatment plans addressing psychological, physiological, and social dimensions based on patient needs. Enhance service satisfaction and management effectiveness through patient feedback mechanisms.

Second, implement community engagement and tiered care systems. Centered on primary healthcare facilities, form teams comprising general practitioners, nurses, and counselors to provide contracted family doctor services. Regular follow-ups monitor patients’ blood glucose, blood pressure, and mental health parameters, with remote coordination with higher-level hospitals for medication adjustments. Mental health service centers in communities provide counseling and support to chronic disease patients, alleviating psychological stress and reducing the incidence of psychiatric comorbidities.

Additionally, digital health tools can augment health management platforms for patients with comorbidities. Leveraging telemedicine, app-based monitoring, and personalized push notifications enables cross-regional and cross-temporal management. Patients use the platform for self-assessment via psychological scales, behavioral/lifestyle guidance, and medication reminders. The platform delivers real-time risk alerts based on monitoring and self-assessment data. Regular remote follow-ups by doctors/nurses and health education articles pushed by the platform enhance self-management awareness and ensure treatment adherence. This approach benefits populations with limited transportation access or low motivation for medical visits, expanding coverage of comprehensive health management services.

**Figure 1:**
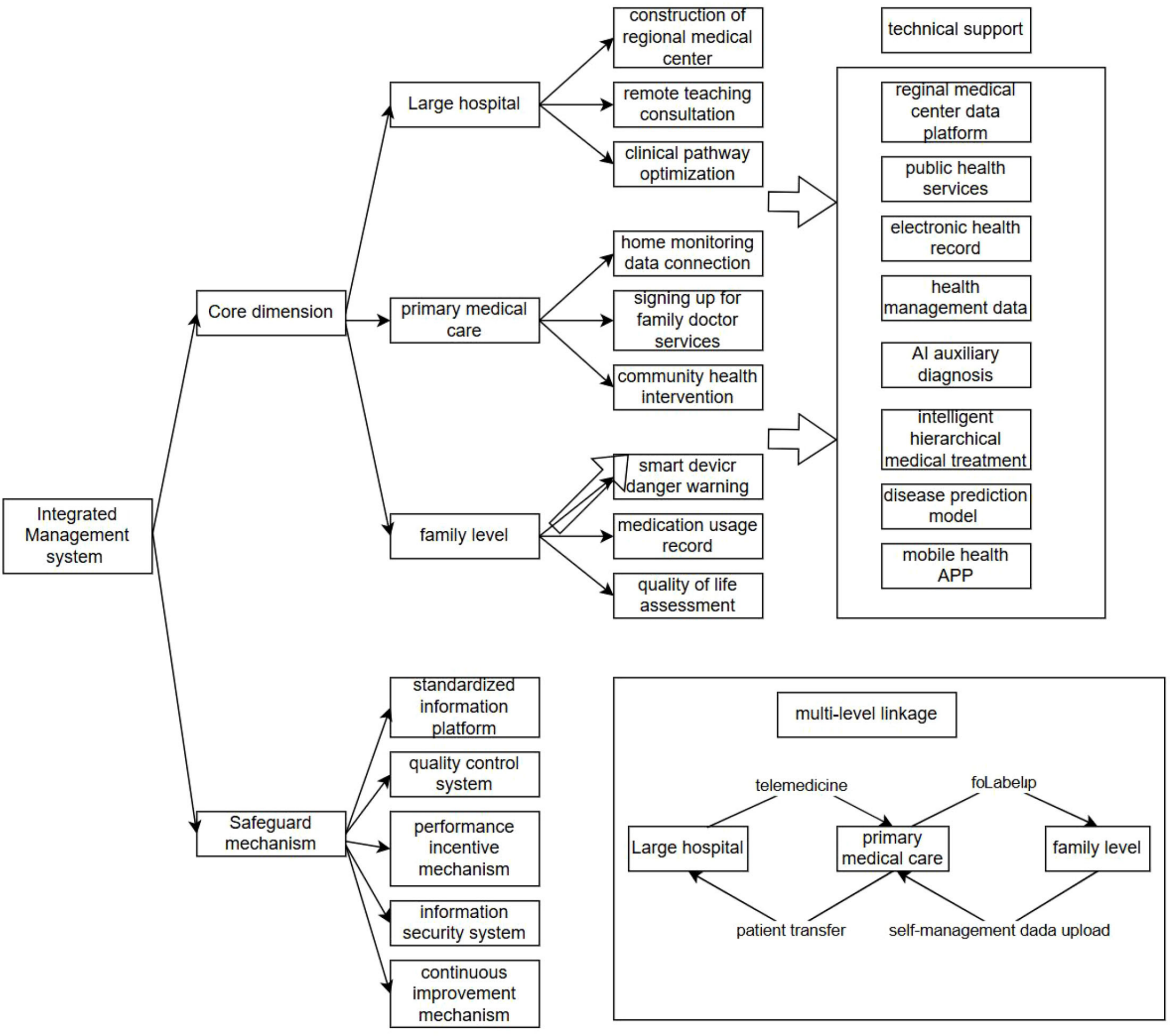
Multi-level management model for patients with comorbidities.

The multi-level management model is shown in **Figure 1**.

## 5 Limitations and future directions

This narrative review has several limitations. First, it did not employ a systematic literature search or formal quality assessment of included studies, which may introduce selection bias. Second, the cited evidence is heterogeneous, encompassing observational studies, clinical trials, and preclinical research, limiting direct comparability. Third, while bidirectional causality is strongly suggested, disentangling the precise temporal and mechanistic sequences in humans remains challenging, and some cited associations should be interpreted as correlational rather than definitively causal.

Future research should prioritize: Longitudinal mechanistic studies to clarify causal pathways, particularly the role of brain insulin resistance and specific inflammatory mediators; High-quality randomized controlled trials (RCTs) evaluating the long-term efficacy and cost-effectiveness of integrated care models on both metabolic and psychiatric outcomes; Implementation science research to identify and overcome barriers to routine metabolic monitoring in psychiatric settings and mental health screening in diabetes clinics; Development and validation of precise biomarkers for predicting comorbidity risk and treatment response.

## 6 Discussion

This review highlights five immediate clinical priorities:(1) Routine metabolic monitoring for patients receiving SGAs;(2) Systematic screening for depression, anxiety, and diabetes distress in diabetes clinics;(3) Early lifestyle and CBT-based psychosocial interventions;(4) Proactive metabolic mitigation when high-risk psychotropics are used;(5) Implementation of collaborative, multidisciplinary care pathways.

Mechanism-to-intervention mapping suggests that inflammatory and metabolic dysregulation may be partially addressed through structured diet and exercise programs; HPA-axis-related stress vulnerability may respond to CBT, sleep regulation, and stress-management approaches; and medication-induced metabolic burden requires monitoring and pharmacologic mitigation. However, uncertainties remain regarding temporal causality, biomarker specificity, and long-term cost-effectiveness of integrated models. Implementation barriers include workforce shortages, data fragmentation, stigma, and digital inequity. Addressing these will require coordinated policy and reimbursement reform.

## 7 Conclusion

In summary, the comorbidity of diabetes and mental disorders arises from a complex interplay of biological, behavioral, and healthcare system factors. This review has synthesized evidence on key mechanistic pathways—including neuroendocrine dysregulation, chronic inflammation, and neurotransmitter abnormalities—and highlighted modifiable risk factors such as lifestyle, medication choices, and fragmented care delivery. Proposed intervention strategies encompass psychometabolic risk screening, combined lifestyle-psychosocial interventions, individualized pharmacotherapy, and integrated care models supported by digital health tools.

Three key messages emerge from this review: (1) comorbidity is the rule rather than the exception, necessitating routine dual screening for mental health symptoms in diabetes settings and metabolic monitoring in psychiatric settings; (2) shared biological pathways inform holistic interventions—targeting inflammation through diet and exercise, regulating HPA axis dysfunction through stress management and CBT, and mitigating medication-induced metabolic burden through careful pharmacovigilance; (3) system-level integration through multidisciplinary teams and digital health platforms is essential for improving clinical outcomes and reducing healthcare disparities.

Addressing existing research gaps through targeted mechanistic studies and implementation science will be crucial to advancing the field and alleviating the dual burden faced by patients with this complex comorbidity.
